# Supplementary material for: The burden of premature adult mortality associated with lack of access to electricity in India
Source: PLoS One. 2024 Mar 13;19(3):e0297463. doi: 10.1371/journal.pone.0297463 (PMC10936845; doi:10.1371/journal.pone.0297463)
Supplement: Attachment — Submitted filename: Response to Reviewers.docx [file pone.0297463.s002.docx]

**Response to Reviewers**

**Reviewer #1:**

**COMMENT 1: An interesting subject has been notified in this manuscript; however, data has been collected in 2014 (9 years ago). It is recommended to use the verbs in past tense, not present, in "Methods" and also in the last paragraph of "Introduction" section (we performed).**

**RESPONSE:** We thank the reviewer for reviewing our manuscript and providing these valuable comments. As suggested, we have combed through the manuscript to ensure the tense of our verbiage is in the past to conform to the timeline of data collection.

**COMMENT 2: "Premature Death" should be defined, before it has been reported in "Results".**

**RESPONSE:** We have now defined premature death and our outcomes measures in the Methods section in pages 3 & 4.

**‘***Premature mortality was defined as the death of an individual aged 15 years and above who died before reaching the age of 70 years.* Based on the 2011 census, the country of India was divided into 640 districts for which district-level estimates were derived from various publicly available data sources.**’**

‘Two separate outcome measures were included in our analysis. District-level age-standardized premature mortality rate estimated in 2014 was obtained by dividing district deaths by population. Conditional probability of premature death was defined as the probability that an individual aged 15 years would die before reaching the age of 70 years. Both outcome measures were derived through rigorous methods employed by Ram et al. and made publicly available.’

**COMMENT 3: Please check the "Keywords" according to main heading terms of the MeSH.**

**RESPONSE:** We have made necessary changes to conform to the available MeSH terms

**COMMENT 4: Fig 4 has been inserted in text with citation to this reference: (Irwin BR, Hoxha K, Grépin KA. Conceptualizing the effect of access to electricity on health in low259 and middle-income countries: A systematic review). Please get a written permission from the corresponding author for using this figure.**

**RESPONSE:** Figure 4 is an original figure by the authors, and is not from the citation mentioned. The citation was inserted in a different context, and has now been removed to avoid confusion. We have obtained permission for Figure 1, as suggested by the editor.

**Reviewer #2:**

**COMMENT 1: The author did not provide sufficient literature to highlight the issues. More literature is required to find the research gaps.
What are the other factors associated with Pre-mature adult death in India? What are the morbid conditions if lack of electricity access is the only factor? More evidence is required to establish your arguments.**

**RESPONSE:** We appreciate the reviewers’ comments and suggestions to further enhance the description of our work. Accordingly, we have now modified our ‘Introduction’ section to make our case stronger on pages 2 & 3

‘…These contradictory reports of the impact of electrification on surrogates measures of adult mortality warrants defining the direct association between access to electricity and all-cause mortality in a nationwide sample of adults.(3)

In 2014, Ram et al. reported that nearly two-fifths of India’s men and one-third of women had >50% and >40% probability of dying before the age of 70 years, respectively. Importantly, they identified a large district-level survival gap with the highest premature mortality rates in east India and the lowest in west India.(6) Numerous infectious and non-communicable diseases accounted for only 60% of the gap with the remaining unexplained. However, multiple individual socio-economic deprivations such as inadequate household income, literacy and employment rates have been associated with increased premature mortality.(7, 8) Additionally, environmental factors that have a community-wide impact such as water pollution, ambient and household air pollution have further increased rates of premature mortality in low-and-middle income countries such as India.(9, 10) With access to reliable electricity being an indicator of socio-economic development, we sought to understand the effect of lack of electricity access on adult premature mortality, and discuss potential pathways of a causal effect.’

**COMMENT 2: What are the variables abstract from DLHS or Census, 2011-India? The author should give the details about the process of data accessibility. What is the procedure followed to analyze the data?**

**RESPONSE:** We used three separate data sources to derive multiple variables that were needed to complete our analysis. Below is a list of all the variables abstracted from each data source.

| **Data Source** | **Variables Extracted** |
| --- | --- |
| Analysis by Ram et al. published in the Lancet Global 2015 | District Population  Age-standardized premature mortality rates in 2014  Conditional probability of premature death in 2014 |
| 2011 Census India | Use of electricity for household lighting purposes  Female literacy rate  Female work participation rate  District-level percentage of urban population  Use of polluting cooking fuels  Access to treated water source |
| Dalhousie University Global Database | Annual fine PM2.5 |

To enhance clarity, we have now modified and rearranged the ‘Data Sources’ section of the Methods to better describe the variables abstracted from each source on pages 3 &4. Additionally, we have created a separate section that clearly describes our outcome measures on page 4:

‘Outcome Measures:

Two separate outcome measures were included in our analysis. District-level age-standardized premature mortality rate estimated in 2014 which is a point estimate. Conditional probability of premature death was defined as the probability that an individual aged 15 years would die before reaching the age of 70 years. Both outcome measures were derived through rigorous methods employed by Ram et al. and made publicly available.(6)’

Our primary analysis was a multivariable linear regression assessing the relationship between electricity access and premature mortality rates and conditional probability of premature death in 2014. We used the coefficients from the regression analysis to derive the excess number of deaths attributed to electricity access. Additionally, we used correlation tests to visualize the above relationship and plotted it using scatterplots.

We have also reframed the ‘Statistical Analysis’ section to better describe the various analytical procedures employed in this study on pages 4 & 5:

‘We estimated the mortality effects of living in a district with reduced electricity access (middle access and low access districts) using multivariable linear regression models, with district level age-standardized premature mortality rates as the outcome measure in one set of models and conditional probability of premature death as the outcome measure in a separate set of models. Models were developed separately for men and women. To account for confounding of a variety of socio-ecological variables that could confound our exposure-outcome association, we adjusted for the following district-level variables: the total population of men and women in the district, percentage of urban population, female literacy, female employment, households using polluting cooking fuels, access to treated drinking water, and PM_2.5_ concentrations in 2014. The actual number of excess premature deaths attributable to reduced electricity access was estimated using the coefficient of age-standardized premature mortality rates derived from the regression models and the total population living in districts with reduced electricity access. Within reduced electricity access districts, we also estimated the proportion of premature deaths attributable to reduced electricity access using the coefficient of conditional probability of death from the regression model and the mean conditional probability of death in those districts.

Scatterplots were used to visualize the relationship between age-standardized premature mortality rates and electricity access rates in districts. Pearson correlation coefficients were used to measure the strengths of correlation. Lastly, using similar linear regression models, we estimated the change in premature mortality rates and probability of premature death, per 1-standard deviation (SD) of increased access to electricity. We replicated this analysis by replacing electricity access with other more established social determinants of health (increased access to treated water, increased female literacy, and decreased use of polluting cooking fuel indicating household air pollution) as the independent variable. The change in overall premature mortality rates and probability of premature death was compared to show the impact of improving access to electricity. Estimates were made separately for men and women.’

**COMMENT 3: The morbid condition associated with low electricity access is missing from the result section. The author is required to follow the methodology in a rigorous way.**

**RESPONSE:** We did not specifically investigate the diseases that lead from low electricity access to increased mortality. We have, however, highlighted potential pathways from low electricity access to increased mortality in our discussion (third paragraph), and figure 4.

We have also reorganized the description of our results by combining the results of our primary analysis into a single paragraph with a separate subheading under the Results section pages 6 & 7.

‘Premature Mortality Estimates for Women and Men Living in Districts with Reduced (Middle and Low) Electricity Access

Table 2 shows that when compared to districts with high electricity access, middle access districts demonstrated a higher rate of age-standardized premature mortality among women (0.55, 95% CI 0.06 to 1.03, p 0.03), but not among men (-0.23, -0.88 to -0.43, p 0.5). Similarly, there was an increased rate of conditional probability of premature death in women (2.41, 95% CI 0.22, 4.60, p 0.03) but not in men (-0.41, 95% CI -2.82 to 2.01, p 0.74). On average, 45,195 (79,342 in women and -34,147 in men) excess premature deaths were attributable to middle range access to electricity. Compared to high access districts, those with low access had higher rates of age-standardized premature mortality in both women (2.09, 95% CI 1.43 to 2.74, p <0.001) and men (0.99, 95% CI 0.10 to 1.87, p 0.03), and a higher conditional probability of premature death in both women (9.16, 95% CI 6.19 to 12.13, p <0.001) and men (4.04, 95% CI 0.77 to 7.30, p 0.02). On average, 399,030 excess deaths (265,959 women and 133,071 men) were attributable to the low access of electricity. The proportion of premature adult deaths attributable to reduced electricity access was 21.3% (14.4% – 28.1%) among women and 7.9% (1.5% to 14.3%) among men.’

Additionally, Figure 2 and 3 have been renamed to clarify our finding of an inverse relationship between electricity access and premature death.

**COMMENT 4: The author should highlight the key inference of the present study, how this study proved the lack of electric access leads to adult mortality.**

**RESPONSE:** The various findings within our analysis are now summarized in paragraph 1 of the Discussion section on page 8. It highlights the primary results which show that electricity access rates are inversely related to premature mortality among women and men in India with women being more impacted compared to men. Given that this is an observational study, there are limitations to making a strong causal inference. We have noted this under limitations as below,

‘Our findings must be interpreted in the context of several limitations. First, given the observational nature of the study, residual confounding cannot be excluded. Second, we performed a cross-sectional analysis to assess the association between electricity access and premature death and cannot assume causality.’

**Reviewer #3:**

**COMMENT 1: The paper is well-written and succinct, but it would benefit from a mention of the methodology used and the data sources employed in the study.**

**RESPONSE:** We appreciate the valuable feedback provided by the reviewer. As described above in response to Reviewer 2, we have now made several changes to the methods section of the manuscript including ‘Statistical Analysis’ which now improves clarity regarding the various methods used to derive our results.

**COMMENT 2: Additionally, specifying the geographic representation of the chosen districts and discussing policy implications could enhance the paper's completeness.**

**RESPONSE:** We are not sure what is implied by ‘chosen districts’. All districts of India were part of the sample for this study. Districts with low electricity access have been highlighted in a map in figure 1.

The major policy implication is to highlight electricity access as a major social determinant of health, which we have sought to do throughout the results and discussion. As highlighted in the introduction, access to electricity is usually not regarded as a major social determinant of health. We have also highlighted in the discussion and conclusion that along with increasing electricity access, ensuring a reliable, zero-carbon supply will be crucial to realizing the full potential of electrification to enhance human health.

**: In the paper, it's essential to provide more details on the methodology, acknowledge data limitations and potential biases, and address the issue of causality.**

**RESPONSE:** We have updated our section on study limitations to highlight the major limitations of our observational analysis on page 11

‘Our findings must be interpreted in the context of several limitations. First, given the observational nature of the study, residual confounding cannot be excluded. Second, we performed a cross-sectional analysis to assess the association between electricity access and premature death and cannot assume causality. Third, we do not measure the effect of electricity reliability (duration of supply in a day, power outages, voltage fluctuations), which will likely be important to fully realize the beneficial health effects of electricity access. Fourth, given that India’s electricity coverage has increased since the 2011 census, the estimates do not reflect the current burden of mortality.’

**COMMENT 4: Exploring variation within low-access districts and offering insights into the gender disparities observed would strengthen the paper's analysis.**

**RESPONSE:** Low access districts were clustered together into a single cohort based on the definition of those districts that had <50% of its households with access to electricity. We do not have granular data regarding the variation in outcomes within this single cohort of ‘low access districts’.

We have now expanded on our understanding of the reasons behind the observed gender disparities on page 8:

‘We found that lack of electricity access leads to a higher probability of premature mortality in women, compared to men. Electricity access is closely linked to women’s empowerment, and its presence may disproportionately benefit women. Previous studies have shown a strong inverse correlation between the level of electrification and maternal mortality rates.(13) Women are often responsible for kerosene related tasks in the household and may suffer disproportionately from its impact. An observation in Chhattisgarh, India, demonstrated that village electrification resulted in improving flexibility in time use by postponing dinner cooking by ~40 minutes. This was accompanied by a 67% decrease in the use of kerosene. Additionally, several broader effects of electrification on gender norms have been described such as enhanced women’s employment, reduced time spent on drudgery, and improved women’s literacy rates.(14) Furthermore, access to communication systems, a key component of women’s empowerment, is critically dependent on access to reliable electricity.(15)’.
