# Supplementary material for: The burden of premature adult mortality associated with lack of access to electricity in India
Source: PLoS One. 2024 Mar 13;19(3):e0297463. doi: 10.1371/journal.pone.0297463 (PMC10936845; doi:10.1371/journal.pone.0297463)
Supplement: Attachment — Submitted filename: Response to Reviewers_Round 2.0.docx [file pone.0297463.s003.docx]

**Response to Reviewers - Round 2**

1. The authors have addressed most of the points raised by the reviewers in the earlier version. The article is substantially improved.
2. The introduction section should have a paragraph which needs to highlight the electricity situation in India and the concerned study areas.

**RESPONSE:** We thank the reviewers for considering our previous modifications and providing us with further feedback in improving our manuscript. As suggested by the reviewer, we have now restructured our ‘Introduction’ section and included a new paragraph that clearly describes the rationale behind our aim to understand the association between access to electricity and premature death among Indians. Changes have been made to page 2 as described below:

‘India proposes an ambitious goal of becoming a global economic powerhouse by 2030, largely on the backbone of its relatively young population.(3) Efforts to preserve this young workforce involves combating the numerous individual socio-economic and environmental deprivations that impact premature mortality.(4-7) Access to reliable electricity has been linked to economic growth, leading successive Indian governments over the past two decades to take large strides in improving access to electricity from ~85% in 2014 to ~97% in 2020.(8, 9) However, there appears to be persistent regional variation in access to electricity, its reliability, and satisfaction among users.(10) Moreover, the health implications of expanded electricity access is unclear, particularly given that a lot of electricity is currently generated by fossil fuels. Additionally, there seems to be contradictory data regarding the impact of electrification on the health of populations living in low-and-middle income countries, largely driven by analyses using outcome measures that are surrogate to mortality. Hence, it is important to understand the impact of these governmental efforts to improve electrification on premature mortality of Indians.’’

3. Why the issue is so important to be enquired need to be justifies in the introduction section.

**RESPONSE:** The restructuring of our Introduction section and the inclusion of additional information as mentioned in Comment 1 enhances the justification for studying the issue of electricity.

4. Further, justification of national database from 2014 is needed as it is one decade old and  a new government is in place In India.
5. Why not recent data needs to be explained.

**RESPONSE:** A key part of our analysis was the requirement of data from the Indian Census. The most recent census data available is from 2011 with the 2021 census being postponed. This is the primary reason for using a database from 2014, which is based on the 2011 census. However, given the goal of our analysis is to merely understand the link between electricity access and premature death, we find that a cross sectional analysis at any time point should provide the necessary information. To ensure completeness, we have mentioned this to be a limitation in the Discussion section on page 11.

‘…Fourth, given that India’s electricity coverage has increased since the 2011 census, the estimates do not reflect the current burden of mortality. However, given the postponement of the 2021 census, more recent data is not available.**’**

6. Discussion can compare some of the studies of global south if data is available.
7. Overall the study provide an important message which needs to be presented in a story telling manner.

**RESPONSE:** Data from countries in the global south with regards to this topic is unfortunately limited. References 11-13, which are mentioned and explained in the introduction, summarize the prior literature on this issue. The paucity of data globally on the relationship between premature mortality and access to electricity was the primary motive behind preparing this manuscript.
